# m6A-Related lncRNAs Are Potential Biomarkers for the Prognosis of Metastatic Skin Cutaneous Melanoma

**DOI:** 10.3389/fmolb.2021.687760

**Published:** 2021-05-05

**Authors:** Suyang Huang, Shanshan Lyu, Zhe Gao, Weifeng Zha, Ping Wang, Yunyun Shan, Jianzhong He, Yang Li

**Affiliations:** ^1^ Department of Dermatology, The Third People’s Hospital of Hangzhou, Hangzhou, China; ^2^ Department of Pathology, Guangdong Provincial People’s Hospital, Guangdong Academy of Medical Sciences, Guangzhou, China; ^3^ Department of Pathology, The Fifth Affiliated Hospital, Sun Yat-Sen University, Zhuhai, China

**Keywords:** prognostic biomarkers, lncRNAs, metastatic skin cutaneous melanoma, LASSO regression, N6-methylandenosine

## Abstract

**Background:** The incidence of skin cutaneous melanoma (SKCM) has risen more rapidly than any other solid tumor in the past few decades. The median survival for metastatic melanoma is only six to nine months and the 5°years survival rate of patients with conventional therapy is less than 5%. Our aim was to reveal the potential molecular mechanism in m6A modification of lncRNA and provide candidate prognostic biomarkers for metastatic SKCM.

**Methods:** lncRNAs expression level was obtained by re-annotation in TCGA and CCLE datasets. m6A-related lncRNAs were selected though correlation analysis. Univariate cox regression analysis was used to screen out independent prognostic factors. LASSO Cox regression was performed to construct an m6A-related lncRNA model (m6A-LncM). Univariate survival analysis and ROC curve were used to assess the prognostic efficacy of this model and candidate lncRNAs. Enrichment analysis was used to explore the candidate genes’ functions.

**Results:** We obtained 1,086 common m6A-related lncRNAs after Pearson correlation analysis in both two datasets. 130 out of the 1,086 lncRNAs are independent prognostic factors. 24 crucial lncRNAs were filtered after LASSO Cox regression analysis. All the m6A-LncM and the 24 lncRNAs were related to overall survival. Stratified survival analysis of m6A-LncM showed that the model retains its prognostic efficacy in recurrence, radiation therapy and other subgroups. Enrichment analysis also found that these lncRNAs were immune associated.

**Conclusion:** Here, we obtained 24 crucial lncRNAs that may be potential biomarkers to predict survival of metastatic SKCM and may provide a new insight to improve the prognosis of it.

## Introduction

Based on the anatomical location, melanomas could be subdivided into limbic melanoma, skin cutaneous melanoma (SKCM), and mucosal melanoma (Curtin et al., 2005; Curtin et al., 2006), of which skin cutaneous melanoma is the major subtype of melanoma in Caucasians and the proportion of SKCM is roughly 20% of the Asian population (Chang et al., 1998; Chi et al., 2011). The incidence of SKCM has risen more rapidly than any other solid tumor in the past few decades (Eggermont et al., 2014). In recent years, researchers have made significant progress in understanding the biology, genetics, and treatment of SKCM. However, the prognosis remains poor due to the high rate of invasion and metastasis (Bsirini and Smoller, 2018). SKCM can metastasize extensively to the skin, subcutaneous, lymphatic system, lungs, and other non-pulmonary organs, so the patients with metastasis have poor survival rates and metastasis is also a major obstacle to improving prognosis (Balch et al., 2009; Domingues et al., 2018; Kremenovic et al., 2020). Therapies such as neoadjuvant treatment and targeted therapy have been shown to improve the prognosis of metastatic melanoma to some extent (Bai et al., 2019). The median survival for metastatic melanoma is only 6 to 9°months and the 5°years survival rate of patients with conventional therapy is less than 5% (Agarwala, 2009). Therefore, the search for effective biomarkers for prediction of prognosis and new therapeutic targets is urgent.

M6A is a mechanism of post-transcriptional modification of RNA prevalent in eukaryotic cells (Chen et al., 2019). m6A modification is involved in the degradation, translation, splicing, and other processes of mRNA (Liu et al., 2017; Chen et al., 2019; Liu and Gregory, 2019). m6A is frequently found in the 3′ UTR stop codon region and exon region, respectively (Dominissini et al., 2012; Meyer et al., 2012). The process of m6A modification is regulated by a variety of relevant factors (Jia et al., 2013). m6A regulators can be divided into three types based on previous research: 1) Writers that can recognize RNA and modify m6A, which includes *KIAA1429*, *METTL3*, *RBM15*, *METTL14*, *WTAP*, *METTL16*, and *ZC3H13* (Balacco and Soller, 2019); 2) Erasers, which are primarily responsible for the removal of m6A modifications, including *FTO* and *ALKNH5* (Liu et al., 2018; Pan et al., 2018) and; 3) Readers, including *HNRNPC*, *HNRNPA2B1*, *YTHDF1*, *YTHDC1*, *YTHDF2*, *YTHDC2*, and *YTHDF3* (Wang et al., 2018). The Readers could identify RNA methylation modifications and participate in RNA translation, degradation, and other processes (Ma et al., 2019). m6A affects many important life processes and is essential for cell division and proliferation, focal death and apoptosis (Zhou et al., 2019). Numerous studies have confirmed that aberrant m6A modifications play a key role in the genesis and development of a variety of tumors, including SKCM (He et al., 2021; Yu et al., 2021). For example, M6A methyltransferase *METTL3* is upregulated in melanoma and modulates melanoma cell invasiveness through *MMP2* (Dahal et al., 2019). *FTO* can promote bladder cancer by regulating the *miR-384*, *MALAT*, *MAL2* axis through m6A modifications (Tao et al., 2021). The *IGF2BP2*, *LINC00460*, and *DHX9* complex could promote colorectal cancer proliferation and metastasis by mediating the stability of *HMGA1* (Hou et al., 2021).

LncRNAs are transcripts with more than 200 nucleotides and with non-coding potential (Ramilowski et al., 2020). The function of lncRNAs remains largely unknown. LncRNA may be involved in the regulation of mRNA expression through translational regulation, histone modifications, or post-transcriptional (Huang et al., 2018). LncRNAs can influence various aspects of tumor cells including survival, proliferation, and migration though participating in gene regulation (Ramilowski et al., 2020). Aberrant expression of lncRNA is associated with tumor malignancy and has been shown to play a key role in the development of numerous cancers including SKCM. For instance, lncRNA *TTN-AS1* promotes SKCM development and metastasis by maintaining *TTN* expression (Wang et al., 2020). LncRNA *HCP5* inhibits the development of SKCM through regulation of *miR-12* expression (Wei et al., 2019).

**FIGURE 1:**
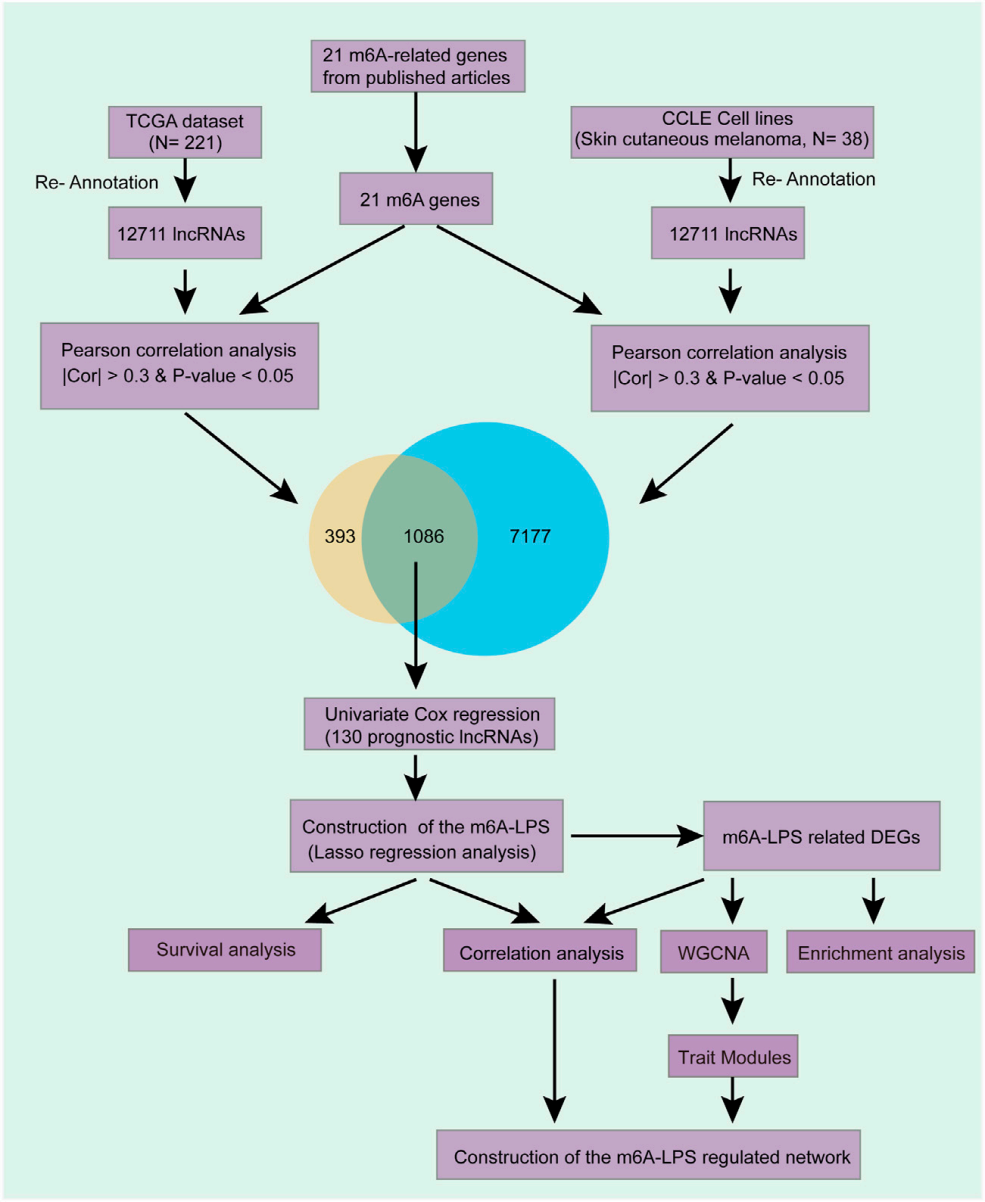
Overview of the comprehensive analysis. We first obtained 12,711 lncRNAs expression level by re-annotation the TCGA and CCLE SKCM data. Then, 1,086 lncRNAs which correlated with at least one of the 21 m6A genes in both datasets after correlation analysis were filtered. Then, 130 out of the 1,086 lncRNAs are independent prognostic factors after Univariate Cox regression and had been selected for further analysis. We used LASSO Cox regression analysis to further filter a model that contained 24 crucial lncRNAs and have an excellent prognostic efficacy. Stratified survival analysis further testified the model’s prognostic efficacy. Finally, enrichment analysis, WGCNA, correlation analysis were used to explored these lncRNAs’ potential function in SKCM.

In this article, we identified m6A modifications related lncRNAs and explored their prognostic ability by bioinformatic analysis and finally obtained potential biomarkers which can predict SKCM prognosis. We also established an important m6A related lncRNA-mRNA regulatory network to provide a new insight to investigate the mechanism of SKCM (Figure 1).

## Materials and Methods

### Ethical Compliance

Public data was used for this study and there are no ethical issues.

### Data Sources

Expression data of 484 SKCM patients and corresponding clinical characteristics were obtained from the GDC data portal using gdc-client (https://gdc-portal.nci.nih.gov/). As described in the Introduction, SKCM has a high rate of metastasis and a poor survival rate of patients with metastasis. Thus, our goal is developing the prognostic biomarkers that could predict the poor survival of metastatic SKCM. Then, we filtered 221 patients who with metastatic status for further analysis (Table 1). We also extracted the expression profile of 38 SKCM cell lines through CCLE database. All the gene expression profiles were quantified by FPKM and normalized though log2-based transformation. In addition, the expression level of 21 m6A-related genes (*FTO, ALKBH5, RBM15, RBM15B, METTL3, METTL14, METTL16, WTAP, VIRMA, ZC3H13, HNRNPC, HNRNPA2B1, IGF2BP1, IGF2BP2, IGF2BP3, RBMX, YTHDC1, YTHDC2*, *YTHDF1*, *YTHDF2*, and *YTHDF3*) (Zaccara et al., 2019) were constructed from the two datasets, respectively.

**TABLE 1:** Clinicopathological characteristics of 221 Metastatic SKCM patients in TCGA dataset.

Clinical and pathological indices	Case No.	OS (%)	*p* value^a^
Specimens	221		
Mean age	57		
Age (years)			<0.001
≤57	111	43.2	
>57	110	34.5	
Gender			0.272
Male	139	36.0	
Female	82	43.9	
Pathologic stage			<0.001
I	62	56.5	
II	41	29.3	
III	85	41.2	
IV	10	40.0	
NA	23		
History of neoadjuvant treatment			0.881
Yes	15	40.0	
No	206	38.8	
Recurrence			<0.001
Yes	146	23.3	
No	75	69.3	
Radiation therapy			0.043
Yes	23	65.2	
No	198	35.9	

a Log-rank test using the Kaplan Meier method; *p* value < 0.05 was considered significant.

### Re-Annotation of lncRNAs

Annotation information of lncRNAs were downloaded from GENECODE (https://www.gencodegenes.org/human/) and the genome annotation file (GRCh38) was downloaded from the UCSC database (http://hgdownload.cse.ucsc.edu/). Based on the annotation information and Ensemble IDs, we obtained 12,711 lncRNAs in TCGA dataset and CCLE dataset.

### Correlation Analysis Between lncRNAs and m6A-Related Genes

Pearson analysis was used to evaluate the correlation between these genes and lncRNAs based on the expression level of lncRNAs and the 21 m6A-related genes. LncRNAs with *p* value < 0.05 and an absolute Pearson correlation coeffcient (PCC) ≥ 0.3 were selected as m6A-related lncRNAs. We then also used Spearman correlation to double-check the correlation (*p* value < 0.05, absolute correlation coefficient ≥0.3). LncRNAs that were significantly associated with at least one of the 21 m6A genes in both data sets were selected for subsequent analysis.

### Predict the Interactions Between lncRNAs and m6A Regulators and Predict m6A Modification Sites of lncRNAs

RNAInter (RNA Interactome Database) was used to predict the interactions between these lncRNAs and m6A regulators based on the RNA sequence (Lin et al., 2020). SRAMP database was used to predict m6A modification sites of the lncRNAs (Zhou et al., 2016).

### Univariate Cox Regression Analysis and Lasso Analysis

The univariate cox regression analysis was used to screen out the prognostic lncRNAs. Subsequently, the R package *glmnet* (Friedman et al., 2010) was used to construct an m6A-related lncRNA prognostic model (m6A-LncM) of SKCM patients by LASSO Cox regression. The riskscore of the LASSO regression model could be calculated as follow:

$$\text{Riskscore}=\sum_{i=1}^{n}\beta_{i}*E_{i}$$

where *E*_*i*_ is the expression value of the *i* gene in the model, and *βi* is the coefficient calculated by LASSO.

### Differential Expression Analysis

Based on the riskscore of m6A-LncM, we classified the 221 SKCM patients into high or low risk scores groups. Then, differential expression analysis between the high and low groups was performed using the *limma* R package. The differentially expressed genes (DEGs) were identified with an adjusted *p* value < 0.05 and an absolute log2 fold change ≥0.585 (1.5 fold change).

### Survival Analysis

Kaplan-Meier analysis was used to evaluate the prognostic efficiency of m6A-LncM and candidate lncRNAs. Survival curves reflect the relationship between the survival model or lncRNA expression level and SKCM patients’ survival status though the survival R package. The *pROC* package was used to calculate the area under the ROC curve (AUC) of the m6A-LncM to assess the prognostic efficiency of it. We also compare the overall survival information between different subgroups of SKCM patients based on the riskscore of the model. The subgroups separated by the following features: age (≤57 or >57°years), neoadjuvant treatment (Yes or No), gender (male or female), melanoma clark level (I, II, III or IV, V), Recurrence (Yes or No), pathologic stage (I, II or III, IV), neoplasm cancer status (with tumor of tumor free), radiation therapy (Yes or No), and tumor issue site (regional or distant). *Caret* R package was used to randomly separate the 221 samples in two self-dependent test datasets (1:1) which were used to testify the survival efficacy of these lncRNAs.

### WGCNA Network Construction and Module Identification

First, the co-expression network of m6A-LncM-related DEGs was constructed by an automatic network construction function in *WGCNA* R package. Second, co-expression modules were detected by the hierarchical clustering function. Then, modules were associated with clinical characteristics by calculating gene significance (GS) and module membership (MM). Finally, the candidate module with key genes were selected for further analysis.

### Construction of the Co-Expression Network

The co-expression network between m6A related genes, m6A-LncM and riskscore-based DEGs was constructed based on the PCC that calculated by Pearson analysis. Those dysregulated lncRNA-mRNA pairs with an absolute PCC ≥ 0.3 and *p* value < 0.05 were selected to construct the co-expression network.

### Function Enrichment Analysis

All the DEGs in the study were extracted for further functional enrichment by using the *clusterProfile* R package and Metascape software. The lncRNAs’ potential functions were obtained from ImmLnc database (Li et al., 2020). Functions with a false discovery rate <0.05 were selected.

## Results

### Screen m6A-Related lncRNAs in Metastatic SKCM Patients and Cell Lines

A total of 12,711 lncRNAs’ expression levels were re-annotated in 221 SKCM patients and 38 SKCM cell lines. Then, we got the expression levels of 21 m6A-associated genes in the two datasets separately and performed Pearson analysis between the 21 genes and the 12,711 lncRNAs. The lncRNAs which exceed the threshold value (*p* value < 0.05 and |PCC| > 0.3) were defined as m6A-related lncRNAs. Finally, we obtained 1,479 lncRNAs that were significantly associated with m6A in 221 SKCM patients and 8,263 eligible ones in 38 SKCM cell lines. Finally, 1,086 lncRNAs that were significantly associated with at least one of the 21 m6A genes in both data sets were selected for subsequent analysis (Figure 1 and Supplementary Table S1).

### Identification of Potential Prognostic lncRNAs and Construct the m6A-LncM

Combined with clinical information, we used univariate Cox regression analysis to filter independent prognostic lncRNAs from the 1,086 lncRNAs related to m6A (*p* value < 0.05). We obtained 130 out of the 1,086 lncRNAs which were significantly associated with overall survival (OS) of metastatic SKCM patients (Supplementary Table S2).

**FIGURE 2:**
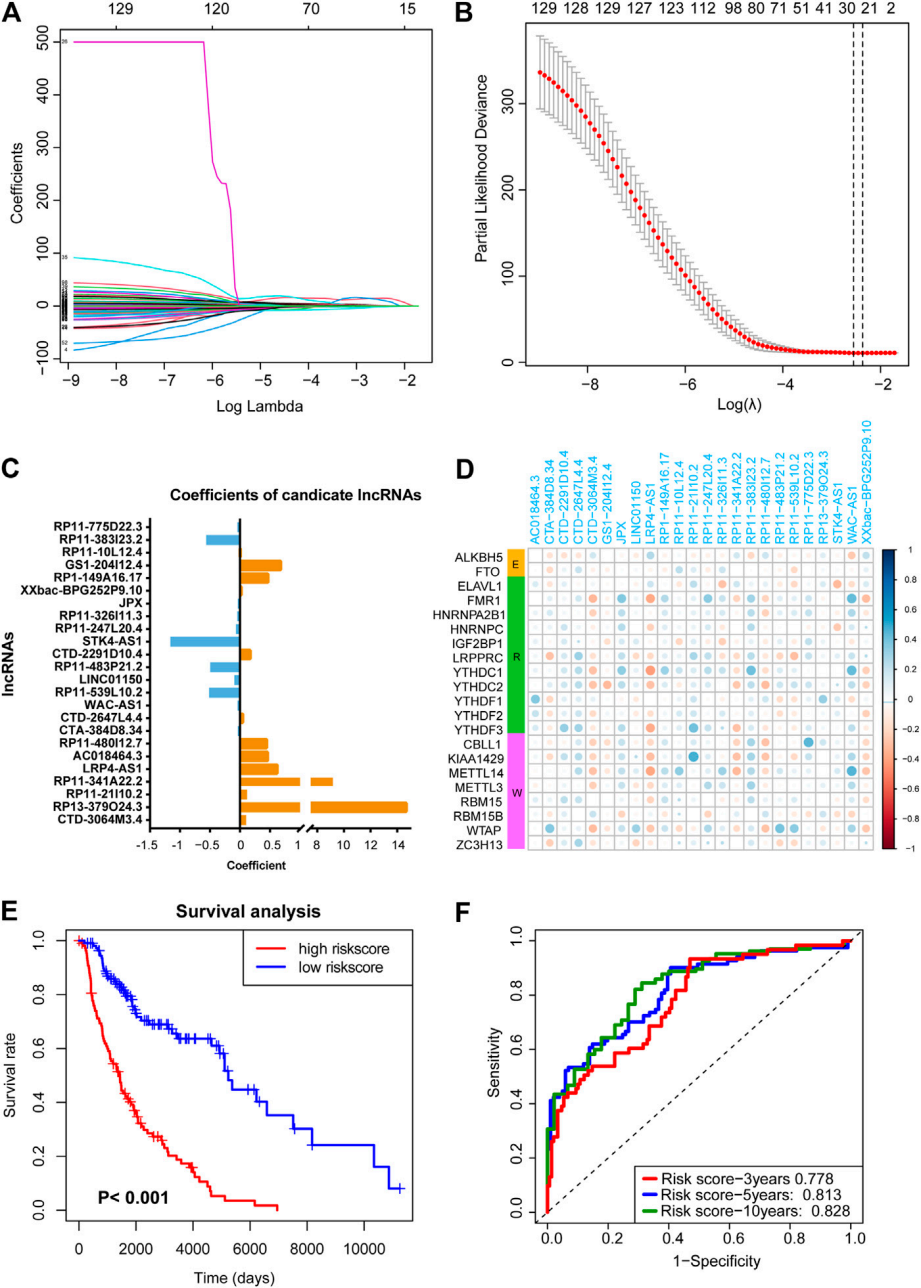
LASSO regression analysis to find crucial survival model and lncRNAs. **(A,B)** The minimum criteria calculated though LASSO regression. **(C)** The coefficients of the survival model constructed by LASSO. **(D)** Heatmap showed the 24 prognostic m6A-related lncRNAs were correlated to the 21 m6A-related genes. **(E)** Survival curves hinted that the subgroup with highly risk score had worse OS rates compare with the low-risk subgroup. **(F)** AUC of the ROC analysis showed the predicted efficacy of m6A-LncM.

Next, LASSO analysis was used on the 130 prognostic lncRNAs to generate an m6A-associated lncRNA model (m6A-LncM) which contains 24 lncRNAs (Figures 2A,B). The risk score of each sample in the dataset was calculated based on the coefficient of the 24 lncRNAs (Figure 2C). The 24 lncRNAs’ PCC were showed in Figure 2D. We then also used Spearman correlation to double-check the correlation. The result showed that all the 24 lncRNAs were significantly associated with at least one of the 21 m6A genes (*p* value < 0.05, absolute correlation coefficient ≥0.3) (Supplementary Figure S1A and Table 2).

**TABLE 2:** Basic information of 24 candidate lncRNAs.

Symbol	ENSG ID	Correlation with m6A regulators Zaccara et al. (2019) (Pearson)	Correlation with m6A regulators (Spearman)	Lasso model coefficients	Survival analysis	
					Role	*p* value
LRP4-AS1	ENSG00000247675	YTHDC1, METTL14, FMR1, YTHDF3	YTHDC1, FMR1	0.619	Risky	1.90E-07
CTD-3064M3.4	ENSG00000244998	FMR1, WTAP	FMR1	0.088	Risky	3.43E-05
RP11–341A22.2	ENSG00000225564	METTL14	METTL14	9.173	Risky	0.0041
STK4-AS1	ENSG00000227477	ELAVL1	ELAVL1	−1.148	Protective	0.0045
GS1-204I12.4	ENSG00000261729	YTHDC2	YTHDC2	0.679	Risky	0.00015
XXbac-BPG252P9.10	ENSG00000272273	FMR1	FMR1	0.019	Risky	0.0038
RP11–480I12.7	ENSG00000234996	YTHDC2	YTHDC2	0.444	Risky	0.0070
RP1-149A16.17	ENSG00000241954	YTHDC1	YTHDC1	0.466	Risky	0.032
RP11–326I11.3	ENSG00000271646	METTL14	METTL14	−0.034	Protective	0.0021
LINC01150	ENSG00000229671	WTAP	WTAP	−0.081	Protective	0.00017
CTD-2647L4.4	ENSG00000259366	LRPPRC	LRPPRC, ZC3H13	0.045	Risky	0.00013
CTD-2291D10.4	ENSG00000267886	YTHDF3	YTHDF3	0.170	Risky	1.52E-07
RP13-379O24.3	ENSG00000272050	YTHDF1	YTHDF1	14.678	Risky	0.0038
RP11-10L12.4	ENSG00000246560	METTL14	METTL14	0.005	Risky	0.0065
JPX	ENSG00000225470	YTHDC1, FMR1	YTHDC1, FMR1, METTL14	−0.023	Protective	0.0062
RP11–247L20.4	ENSG00000259071	FMR1	FMR1	−0.056	Protective	8.00E-04
RP11–539L10.2	ENSG00000246526	WTAP	WTAP	−0.502	Protective	5.57E-05
AC018464.3	ENSG00000234520	YTHDF1	YTHDF1, YTHDF2	0.460	Risky	0.0018
CTA-384D8.34	ENSG00000273272	WTAP	WTAP, LRPPRC	−0.010	Protective	5.56E-06
RP11–483P21.2	ENSG00000260228	WTAP	WTAP	−0.484	Protective	6.72E-05
RP11–21I10.2	ENSG00000249356	YTHDF3, KIAA1429	YTHDF3, KIAA1429,LRPPRC	0.105	Risky	0.00015
RP11–383I23.2	ENSG00000273374	YTHDC1	KIAA1429, YTHDC1,CBLL1, HNRNPA2B1,FMR1	−0.552	Protective	0.0043
WAC-AS1	ENSG00000254635	FMR1, YTHDC1,METTL14	FMR1, YTHDC1METTL14	−0.010	Protective	5.01E-05
RP11–775D22.3	ENSG00000240571	CBLL1	CBLL1	−0.031	Protective	0.0059

Then, based on the median risk score, we divided the patients into high- and low-risk score subgroups. The survival curve hinted that patients whose risk scores were high had a worse survival rate (Figure 2E). The ROC curves also hint that the m6A-LncM had a good efficacy to predict overall survival of metastatic SKCM patients (Figure 2F). We also performed multi-variate survival analysis (cox proportional hazard models by combining the expression of the 24 lncRNAs and OS) to testify the survival efficacy of these lncRNAs. The result showed that they remained significant in multivariate survival models (Supplementary Figure S1B).

### The 24 lncRNAs Have m6A Modification Sites and Have Interactions with m6A Regulators

We used RNAInter (RNA Interactome Database) to predict the interactions between these lncRNAs and m6A regulators based on the RNA sequence. We found that all the lncRNAs have interactions with m6A regulators (Supplementary Table S3). On the other hand, we also used SRAMP database to predict m6A modification sites based on the RNA sequences of these lncRNAs. We found that most of the lncRNAs have potential m6A modification sites except RP11-247L20.4 (Supplementary Table S4). The results of the two tools showed that the 24 lncRNAs are regulated by m6A modification and m6A regulators. It also illustrated that the correlation analysis that we performed is credible.

### Survival Analysis of the Twenty-Four lncRNAs in m6A-LncM

**FIGURE 3:**
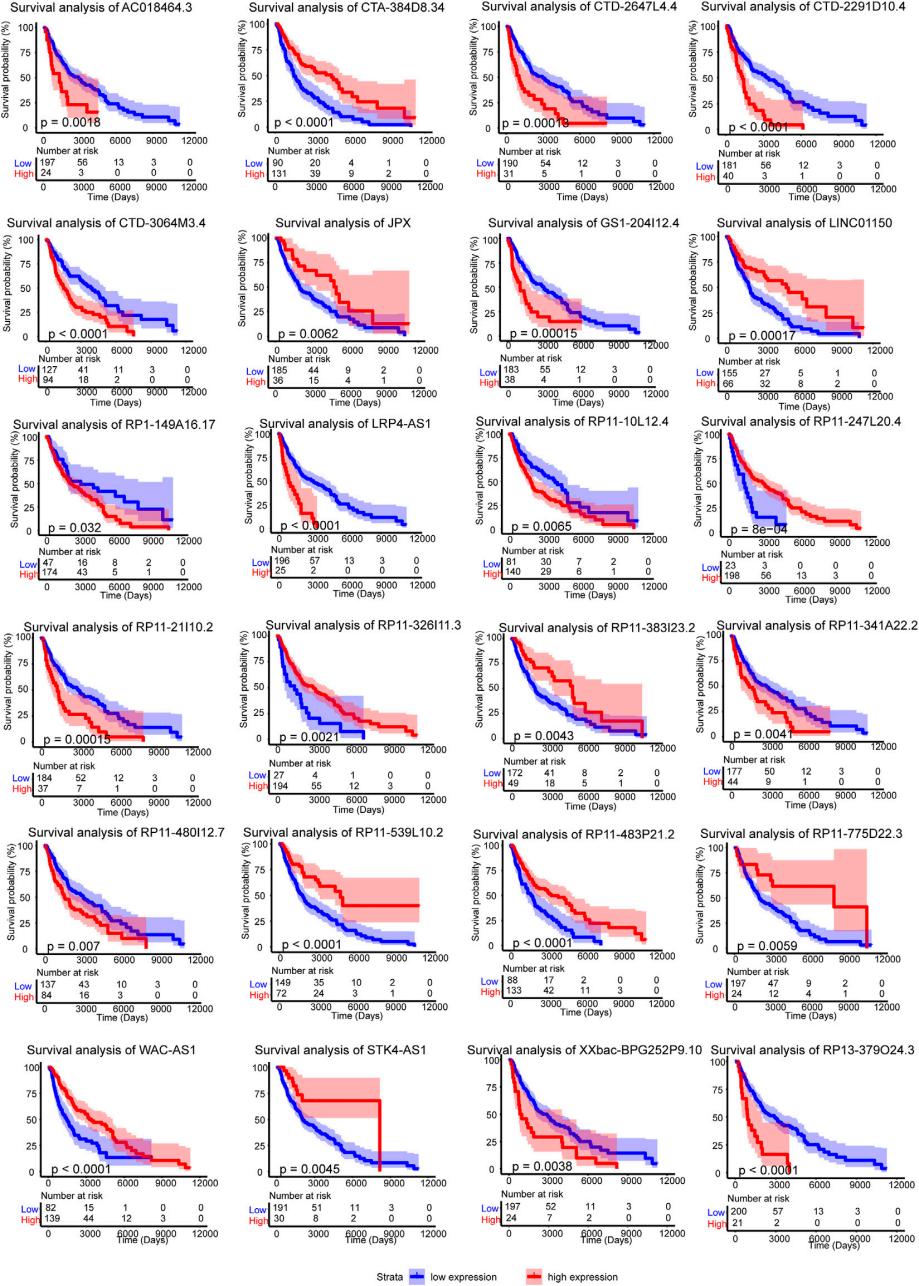
Survival analysis of the 24 crucial lncRNAs in the m6A-LncM. Red line represented the high expression level patients when the blue is low expression. The light color areas represent 95% confidence intervals. The *p* value were calculated by Log-rank test.

The 24 crucial lncRNAs in this model were evaluated by survival analysis. The results showed that all these lncRNAs are survival associated (Figure 3). To be specific, 13 (LRP4-AS1, CTD-3064M3.4, RP11-341A22.2, GS1-204I12.4, XXbac-BPG252P9.10, RP11-480I12.7, RP1-149A16.17, CTD-2647L4.4, CTD-2291D10.4, RP13-379O24.3, RP11-10L12.4, AC018464.3, and RP11-21I10.2) out of the crucial 24 lncRNAs were risk factors of SKCM patients while the other 11 lncRNAs (STK4-AS1, RP11-326I11.3, LINC01150, JPX, RP11-247L20.4, RP11-539L10.2, CTA-384D8.34, RP11-483P21.2, RP11-383I23.2, WAC-AS1, and RP11-775D22.3) are protective factors (Table 2). The two self-dependent test datasets also verified their survival value. Both these 24 lncRNAs have good prognostic efficacy (Supplementary Figures S2, S3).

### Stratified Survival Analysis of the m6A-LncM

**FIGURE 4:**
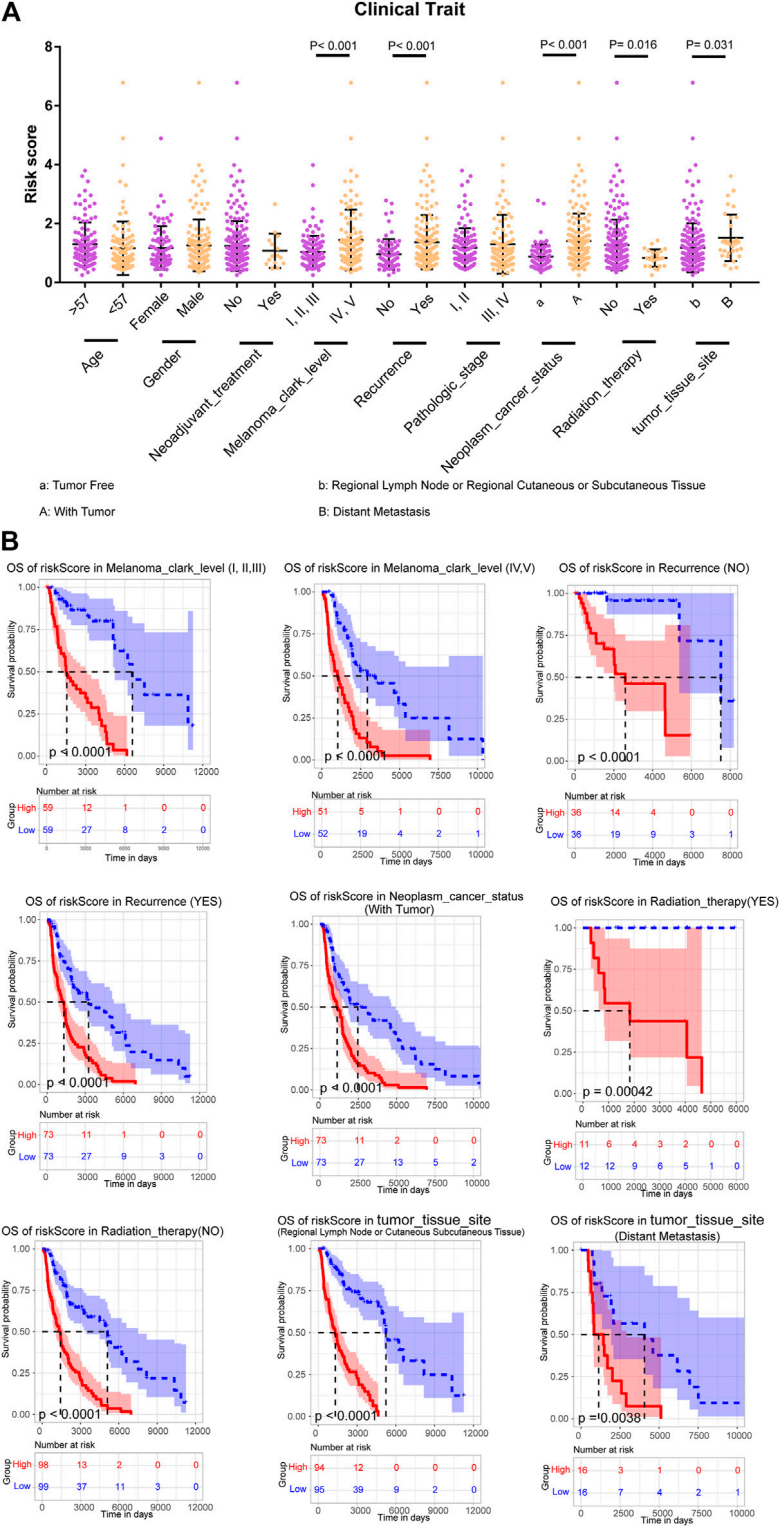
Stratified survival analysis of the m6A-LncM. **(A)** The m6A-LncM could divide patients with different clinicopathological features into differential risk score subgroups. **(B)** The m6A-LncM still could predict the survival in multiple subgroups of metastatic SKCM patients. The red line represented the high riskscore patients when the blue represented low riskscore. The light color areas represent 95% confidence intervals. The *p* value were calculated by Log-rank test.

To further assess the prognostic efficacy of m6A-LncM, we analyzed how the model’s risk scores differed across various clinical traits and found that this model could distinguish melanoma clark level, recurrence, neoplasm cancer status, radiation therapy, and tumor issue site (Figure 4A). Therefore, we performed the stratified survival analysis to explore whether the model could predict OS in these subgroups. Interestingly, the higher risk metastatic SKCM patients had worse survival rate in all the melanoma clark levels (Figures 4B,C). Similarly, patients with high riskscores had a poor prognosis, regardless of recurrence (Figures 4D,E). When the neoplasm cancer status of patients is with tumor, the high riskscore was also associated with a lower survival rate (Figure 4F). We also confirmed that m6A-LncM retained its prognostic efficacy of patients with radiation therapy (Figures 4G,H). Patients with higher risk also had a worse survival rate in both the regional lymph node and distant metastasis subgroups (Figure 4I). These data proved that the model could be a novel predictor and performed an excellent prognostic efficacy in metastatic SKCM and may help to improve the prognosis of this cancer.

### Enrichment Analysis of Key lncRNAs

**FIGURE 5:**
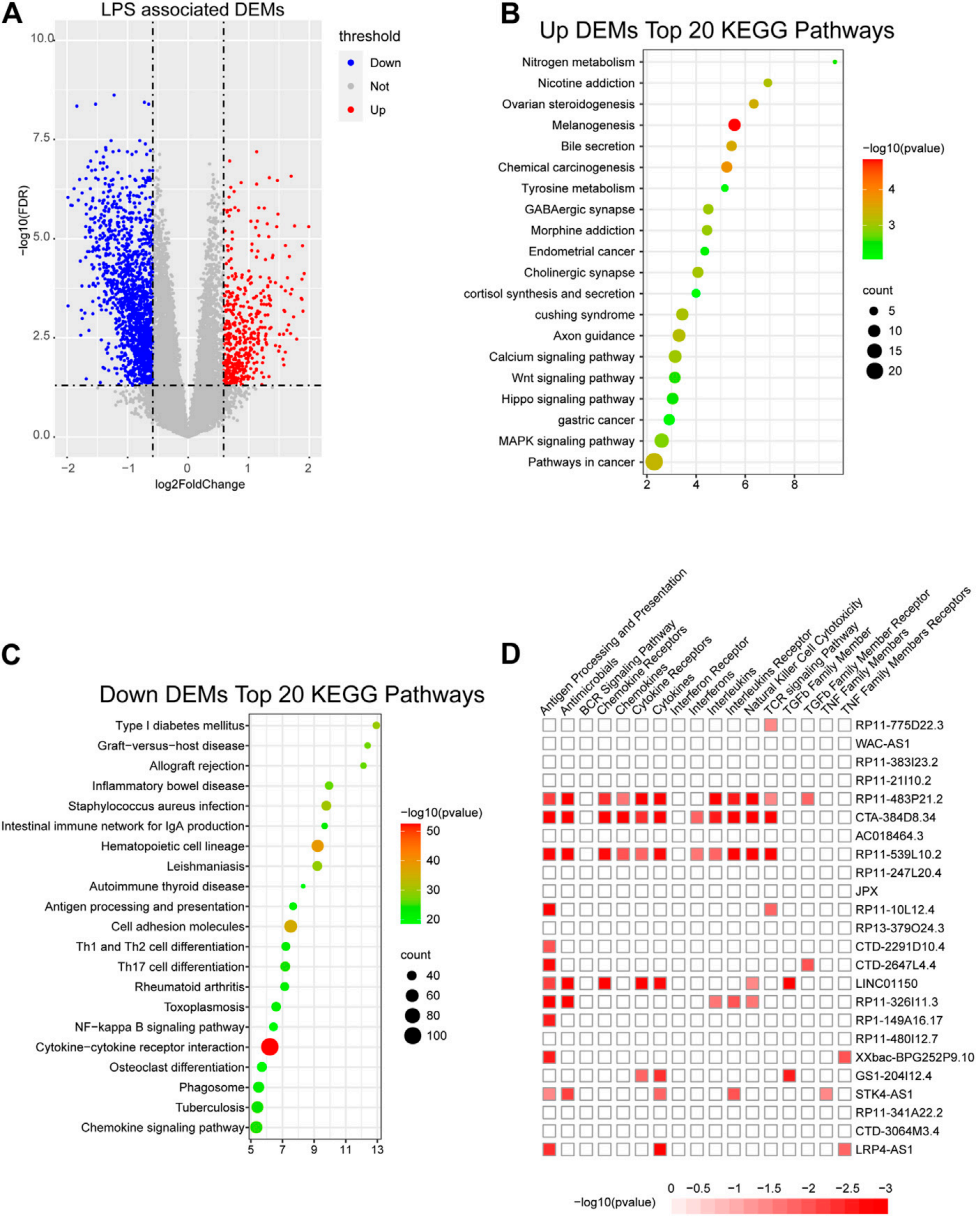
Obtain m6A-LncM associated DEGs. **(A)** Volcano plot shows the differentially expressed genes (DEGs) between high riskscore samples and low riskscore samples. **(B, C)** The functional enrichment analysis of the up-regulated DEGs and down-regulated DEGs. **(D)** The functional enrichment analysis of 24 lncRNAs in m6A-LncM though ImmLnc database.

To further confirm the potential functions of the m6A-LncM and crucial lncRNAs in SKCM, we classified the 221 patients into high- and low-risk score groups based on the model and obtained differentially expressed genes which are regulated by it (Figure 5A). The enrichment analysis showed that the upregulated DEGs were enriched in the Melanogenesis pathway, this further confirms that our model plays an important role in SKCM. Other cancer-related signaling pathways were also enriched, include Calcium signaling pathway, Wnt signaling pathway, Hippo signaling pathway, MAPK signaling pathway, and so on (Figure 5B). The downregulated DEGs were enriched in numerous immune-associated pathways such as Th1 and Th2 cell differentiation, intestinal immune network for IgA production and NF−kappa B signaling pathway (Figure 5C). Based on the result, we conjecture these lncRNAs may play a crucial role in the immune process of SKCM patients. Then, we used ImmLnc database to further testify whether the 24 lncRNAs are related to immune response. As showed in Figure 5D we confirmed these crucial lncRNAs are also enriched in multiple immune-associated pathways. All of these suggest that the m6A-LncM plays an important role in the prognosis of SKCM and the lncRNAs which we obtained also involved in immune response.

### Identification of m6A-LncM-Regulated Gene Modules Associated with Clinical Traits

**FIGURE 6:**
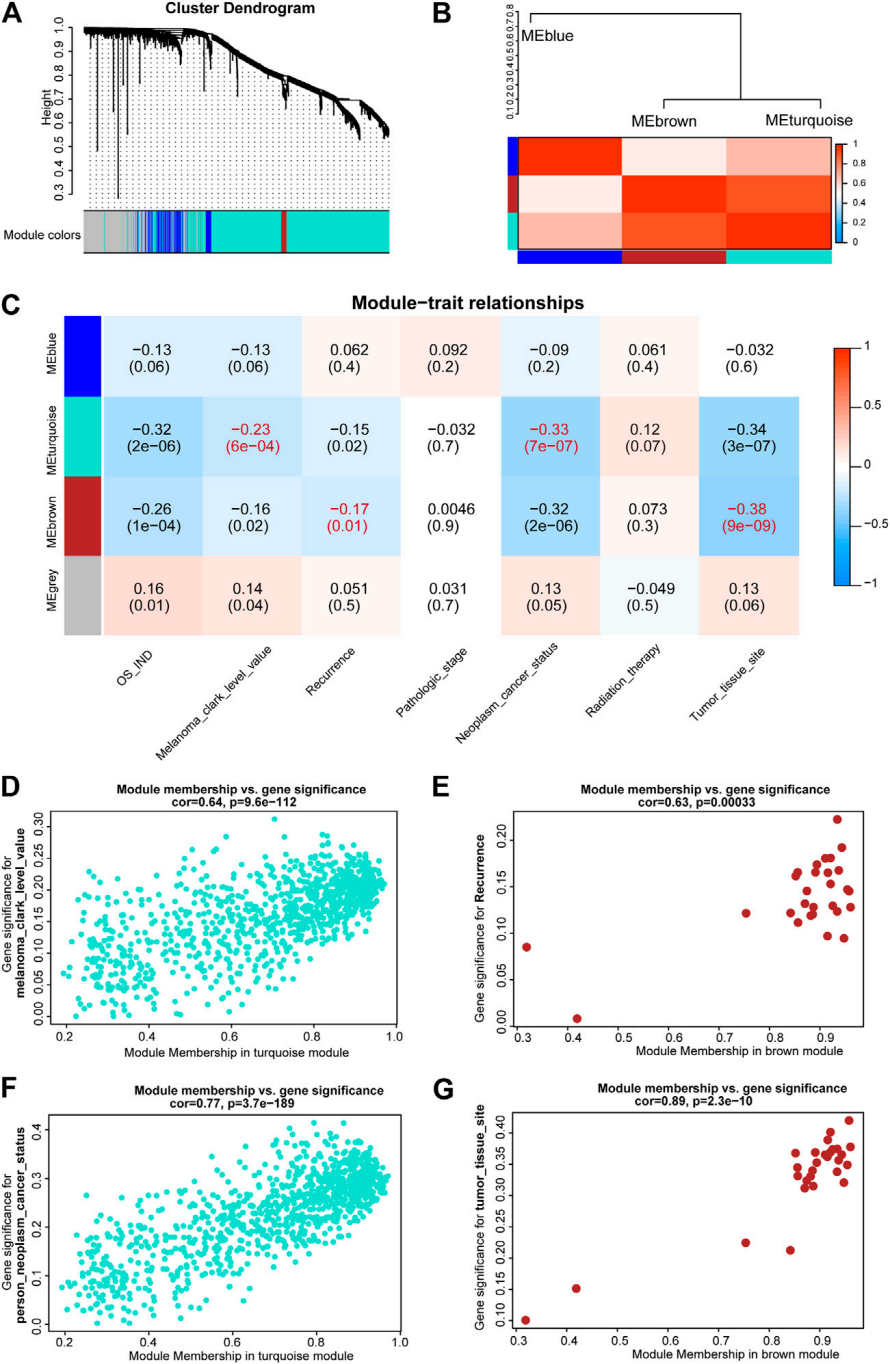
WGCNA of m6A-LncM associated DEGs. **(A)** Clustering dendrogram of genes with dissimilarity based on topological overlap and assigned module colors. **(B)** The connectivity of eigengenes between these modules. **(C)** The relationships between Modules and clinical traits. The *p* value is showed in parentheses. **(D,F)** Scatter plot showed module membership between clinical traits (melanoma clark level and neoplasm cancer status) and turquoize module. **(E,G)** Scatter plot showed module membership between clinical traits (recurrence and tumor issue site) and brown module.

After differential analyses, we selected the m6A-LncM associated DEGs to construct a gene co-expression network by WGCNA. The soft threshold power was set at 6 for further analysis (Supplementary Figure S4). Next, we constructed the gene network and identified modules using the one-step network construction function of the *WGCNA* R package. To cluster splitting, the minimum module size was set at 30 and three modules (blue, brown and turquoize) were generated (Figure 6A). We then analyzed the relationship between these modules. It hinted that there are two clusters over the three modules (Figure 6B).

Subsequently, we correlated modules with patients’ clinical traits to find the crucial genes that associated with clinical characteristics (Figure 6C). The results showed that the turquoize module was significantly associated with melanoma clark level and neoplasm cancer status (Figures 6D,F). The brown module was associated with recurrence and tumor issue site (Figures 6E,G). The blue module was not associated with clinical phenotypes. Finally, we executed an enrichment analysis of the DEGs in the two functional modules and it showed that these key genes were also enriched in immune-associated pathways, such as activation of immune response, alpha-beta T cell activation and lymphocyte migration (Supplementary Figure S5). It may help us to further explore the potential regulatory mechanism of these critical lncRNAs in metastatic SKCM.

### Construct a m6A-Regulated lncRNA-mRNA Co-Expressed Network

**FIGURE 7:**
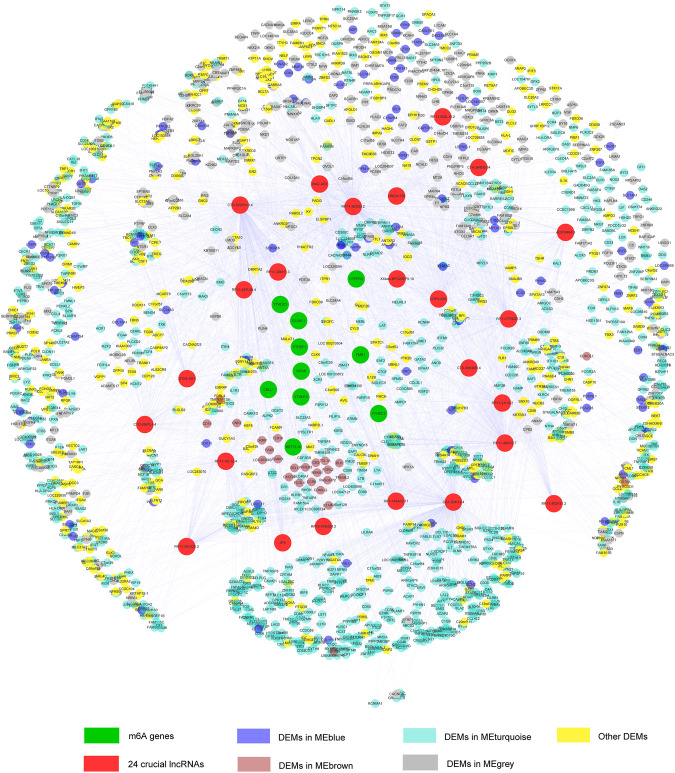
Co-expression network constructed by m6A-related genes, m6A-LncM and DEGs.

To better understand the m6A mediated regulation of lncRNA, we constructed a co-expressed network based on the PCC between the m6A genes, m6A-lncM, and m6A-LncM regulated DEGs. Ultimately, 21 m6Agenes, 24 lncRNAs, and 1,251 mRNAs were selected in the network (Figure 7). The colors of the nodes in the network represent their function in metastatic SKCM.

## Discussion

In recent years, integrate multiple omics analysis has been widely used in cancer research (Chen et al., 2020b). There are also many important studies involved in the prognosis of SKCM. Anjali et al. developed a webserver to predict the survival of SKCM patients (Dhall et al., 2020). Li et al. also predicted the metastatic progression of melanoma based on mRNA expression status (Li et al., 2015). The machine learning model has also been performed to predict primary or metastatic SKCM patients (Bhalla et al., 2019). LncRNAs were also found to play a key role in SKCM. Several studies found that some lncRNAs could distinguish the subtypes of SKCM and predict their survival (Ma et al., 2017; Yang et al., 2018). Overexpressed lncRNA HCP5 could also decrease SKCM cell malignancy *in vitro* which may be upregulated by RARRES3 (Wei et al., 2019). m6A modification has been found that plays an important role in the occurrence and development of tumor. For example, sublethal heat treatment increases EGFR m6A modification near the 5′UTR region and promotes its binding to YTHDF1, thereby enhancing the translation of EGFR mRNA and promoting hepatocellular carcinoma progression (Su et al., 2021). METTL3 dependent m6A modification could regulate the differentiation of T follicle helper cells (Yao et al., 2021). WNT7B-m6A-TCF7L2 positive feedback loop could promote gastric cancer progression and metastasis (Gao et al., 2021). The m6A regulators have also been found by many studies to influence the progression and prognosis of cancers though regulating crucial lncRNAs. For instance, LINC00958 regulated by m6a modification can promote breast cancer tumorigenesis through miR-378a-3p and YY1 axis (Rong et al., 2021). A novel hypoxic long-stranded noncoding RNA KB-1980E6.3 maintains stemness in breast cancer stem cells by interacting with IGF2BP1 to promote the stability of c-Myc mRNA (Zhu et al., 2021). MALAT1 promotes thyroid cancer progression by binding to miR-204 and upregulating IGF2BP2, thereby affecting miR-204/IGF2BP2/m6A-MYC signaling (Ye et al., 2021). In summary, m6A modification of lncRNA is not negligible in tumor progression, however, there are no studies on m6A modification of lncRNAs in SKCM.

Here, we identified a prognostic model including 24 m6A-associated lncRNAs from 221 metastatic SKCM patients. RP11–480I12.5 was reported to promote breast cancer growth and tumorigenesis by inhibiting mir-29c-3p mediated degradation of AKT3 and CDK6 (Lou et al., 2020). LncRNA JPX can promote cervical cancer progression by regulating the miR-25–3p/SOX4 axis (Chen et al., 2020a). These lncRNAs were subsequently found to be closely related to immunity in our study. Interestingly, most of the 24 lncRNAs are novel to cancer research. Compared to the previous lncRNA studies in SKCM, the 24 lncRNAs identified in this study are novel and have the potential for application in clinic. Therefore, we hope that our findings will help to identify potential prognostic lncRNAs regulated by m6A and thus provide ideas to improve the poor prognosis of metastatic SKCM.

### Limitations of the Study

Although we screened several novel m6A-related biomarkers, their specific relationship with m6A modifications needs to be verified experimentally. Meanwhile, due to the lack of lncRNA sequencing data and corresponding clinical information for SKCM, our model was not validated using an independent validation set. Subsequent studies may focus on the development of lncRNA sequencing in SKCM to provide strong evidence for screening more potential biomarkers.

## Conclusion

This is the first study to explore the role of m6A-modified lncRNAs in the prognosis of metastatic SKCM. In this research, we identified the stable correlated m6A-lncRNA pairs in SKCM patients and SKCM cell lines. Then, we constructed a m6A-LncM by lasso regression and found potential prognostic lncRNAs by survival analysis. Twenty-four lncRNAs with independent prognostic efficacy were obtained in the analysis. Next, we comprehensively studied the relationship between these lncRNAs and clinical traits and explored their potential functions. Finally, we delineated a network mediated by these lncRNA, m6A genes and the m6A-LncM regulated differential genes. This study helps to identify potential prognostic targets for metastatic SKCM to improve its poor prognosis.

## Data Availability

The datasets presented in this study can be found in online repositories. The names of the repository/repositories and accession number(s) can be found in the article/Supplementary Material.

## References

[B1] Agarwala S. S. (2009). Current Systemic Therapy for Metastatic Melanoma. Expert Rev. Anticancer Ther. 9 (5), 587–595. 10.1586/era.09.25 19445576

[B2] Bai X. Fisher D. E. Flaherty K. T. (2019). Cell-state Dynamics and Therapeutic Resistance in Melanoma from the Perspective of MITF and IFNγ Pathways. Nat. Rev. Clin. Oncol. 16 (9), 549–562. 10.1038/s41571-019-0204-6 30967646 PMC7185899

[B3] Balacco D. L. Soller M. (2019). The m6A Writer: Rise of a Machine for Growing Tasks. Biochemistry 58 (5), 363–378. 10.1021/acs.biochem.8b01166 30557013

[B4] Balch C. M. Gershenwald J. E. Soong S.-j. Thompson J. F. Atkins M. B. Byrd D. R. (2009). Final Version of 2009 AJCC Melanoma Staging and Classification. Jco 27 (36), 6199–6206. 10.1200/JCO.2009.23.4799 PMC2793035 19917835

[B5] Bhalla S. Kaur H. Dhall A. Raghava G. P. S. (2019). Prediction and Analysis of Skin Cancer Progression Using Genomics Profiles of Patients. Sci. Rep. 9 (1), 15790. 10.1038/s41598-019-52134-4 31673075 PMC6823463

[B6] Bsirini C. Smoller B. (2018). Histologic Mimics of Malignant Melanoma. smedj 59 (11), 602–607. 10.11622/smedj.2018041 PMC6250758 29774360

[B7] Chang A. E. Karnell L. H. Menck H. R. (1998).The National Cancer Data Base Report on Cutaneous and Noncutaneous Melanoma. The American College of Surgeons Commission on Cancer and the American Cancer Society, Cancer 83 (8). 1664–1678. 10.1002/(sici)1097-0142(19981015)83:8<1664::aid-cncr23>3.0.co;2-g 9781962

[B8] Chen X.-Y. Zhang J. Zhu J.-S. (2019). The Role of m6A RNA Methylation in Human Cancer. Mol. Cancer 18 (1), 103. 10.1186/s12943-019-1033-z 31142332 PMC6540575

[B9] Chen X. Yang J. Wang Y. (2020a). LncRNA JPX Promotes Cervical Cancer Progression by Modulating miR-25-3p/SOX4 axis. Cancer Cel Int 20, 441. 10.1186/s12935-020-01486-3 PMC7487936 32943989

[B10] Chen Y. Liao L.-D. Wu Z.-Y. Yang Q. Guo J.-C. He J.-Z. (2020b). Identification of Key Genes by Integrating DNA Methylation and Next-Generation Transcriptome Sequencing for Esophageal Squamous Cell Carcinoma. Aging 12 (2), 1332–1365. 10.18632/aging.102686 31962291 PMC7053602

[B11] Chi Z. Li S. Sheng X. Si L. Cui C. Han M. (2011). Clinical Presentation, Histology, and Prognoses of Malignant Melanoma in Ethnic Chinese: a Study of 522 Consecutive Cases. BMC Cancer 11, 85. 10.1186/1471-2407-11-85 21349197 PMC3056833

[B12] Curtin J. A. Busam K. Pinkel D. Bastian B. C. (2006). Somatic Activation of KIT in Distinct Subtypes of Melanoma. Jco 24 (26), 4340–4346. 10.1200/JCO.2006.06.2984 16908931

[B13] Curtin J. A. Fridlyand J. Kageshita T. Patel H. N. Busam K. J. Kutzner H. (2005). Distinct Sets of Genetic Alterations in Melanoma. N. Engl. J. Med. 353 (20), 2135–2147. 10.1056/NEJMoa050092 16291983

[B14] Dahal U. Le K. Gupta M. (2019). RNA m6A Methyltransferase METTL3 Regulates Invasiveness of Melanoma Cells by Matrix Metallopeptidase 2. Melanoma Res. 29 (4), 382–389. 10.1097/CMR.0000000000000580 30762711

[B15] Dhall A. Patiyal S. Kaur H. Bhalla S. Arora C. Raghava G. P. S. (2020). Computing Skin Cutaneous Melanoma Outcome from the HLA-Alleles and Clinical Characteristics. Front. Genet. 11, 221. 10.3389/fgene.2020.00221 32273881 PMC7113398

[B16] Domingues B. Lopes J. Soares P. Populo H. (2018). Melanoma Treatment in Review. Itt Vol. 7, 35–49. 10.2147/ITT.S134842 PMC5995433 29922629

[B17] Dominissini D. Moshitch-Moshkovitz S. Schwartz S. Salmon-Divon M. Ungar L. Osenberg S. (2012). Topology of the Human and Mouse m6A RNA Methylomes Revealed by m6A-Seq. Nature 485 (7397), 201–206. 10.1038/nature11112 22575960

[B18] Eggermont A. M. Spatz A. Robert C. (2014). Cutaneous Melanoma. The Lancet 383 (9919), 816–827. 10.1016/S0140-6736(13)60802-8 24054424

[B19] Friedman J. Hastie T. Tibshirani R. (2010). Regularization Paths for Generalized Linear Models via Coordinate Descent. J. Stat. Softw. 33 (1), 1–22. 10.18637/jss.v033.i01 20808728 PMC2929880

[B20] Gao Q. Yang L. Shen A. Li Y. Li Y. Hu S. (2021). A WNT7B-m6A-Tcf7l2 Positive Feedback Loop Promotes Gastric Cancer Progression and Metastasis. Sig Transduct Target. Ther. 6 (1), 43. 10.1038/s41392-020-00397-z PMC7851143 33526767

[B21] He F. Yu J. Yang J. Wang S. Zhuang A. Shi H. (2021). m ^6^ A RNA Hypermethylation-Induced BACE2 Boosts Intracellular Calcium Release and Accelerates Tumorigenesis of Ocular Melanoma. Mol. Ther. S1525-0016 (21), 00081-2. 10.1016/j.ymthe.2021.02.014 PMC8178445 33601055

[B22] Hou P. Meng S. Li M. Lin T. Chu S. Li Z. (2021). LINC00460/DHX9/IGF2BP2 Complex Promotes Colorectal Cancer Proliferation and Metastasis by Mediating HMGA1 mRNA Stability Depending on m6A Modification. J. Exp. Clin. Cancer Res. 40 (1), 52. 10.1186/s13046-021-01857-2 33526059 PMC7851923

[B23] Huang Q. Yan J. Agami R. (2018). Long Non-coding RNAs in Metastasis. Cancer Metastasis Rev. 37 (1), 75–81. 10.1007/s10555-017-9713-x 29230620

[B24] Jia G. Fu Y. He C. (2013). Reversible RNA Adenosine Methylation in Biological Regulation. Trends Genet. 29 (2), 108–115. 10.1016/j.tig.2012.11.003 23218460 PMC3558665

[B25] Kremenovic M. Schenk M. Lee D. J. (2020). Clinical and Molecular Insights into BCG Immunotherapy for Melanoma. J. Intern. Med. 288 (6), 625–640. 10.1111/joim.13037 32128919

[B26] Li Y. Jiang T. Zhou W. Li J. Li X. Wang Q. (2020). Pan-cancer Characterization of Immune-Related lncRNAs Identifies Potential Oncogenic Biomarkers. Nat. Commun. 11 (1), 1000. 10.1038/s41467-020-14802-2 32081859 PMC7035327

[B27] Li Y. Krahn J. M. Flake G. P. Umbach D. M. Li L. (2015). Toward Predicting Metastatic Progression of Melanoma Based on Gene Expression Data. Pigment Cel Melanoma Res. 28 (4), 453–463. 10.1111/pcmr.12374 PMC4469521 25847062

[B28] Lin Y. Liu T. Cui T. Wang Z. Zhang Y. Tan P. (2020). RNAInter in 2020: RNA Interactome Repository with Increased Coverage and Annotation. Nucleic Acids Res. 48 (D1), D189–D197. 10.1093/nar/gkz804 31906603 PMC6943043

[B29] Liu N. Zhou K. I. Parisien M. Dai Q. Diatchenko L. Pan T. (2017). N 6-methyladenosine Alters RNA Structure to Regulate Binding of a Low-Complexity Protein. Nucleic Acids Res. 45 (10), 6051–6063. 10.1093/nar/gkx141 28334903 PMC5449601

[B30] Liu Q. Gregory R. I. (2019). RNAmod: an Integrated System for the Annotation of mRNA Modifications. Nucleic Acids Res. 47 (W1), W548–W555. 10.1093/nar/gkz479 31147718 PMC6602476

[B31] Liu Z.-X. Li L.-M. Sun H.-L. Liu S.-M. (2018). Link Between m6A Modification and Cancers. Front. Bioeng. Biotechnol. 6, 89. 10.3389/fbioe.2018.00089 30062093 PMC6055048

[B32] Lou W. Ding B. Zhong G. Yao J. Fan W. Fu P. (2020). RP11-480I12.5-004 Promotes Growth and Tumorigenesis of Breast Cancer by Relieving miR-29c-3p-Mediated AKT3 and CDK6 Degradation. Mol. Ther. - Nucleic Acids 21, 916–931. 10.1016/j.omtn.2020.07.022 32810693 PMC7452110

[B33] Ma S. Chen C. Ji X. Liu J. Zhou Q. Wang G. (2019). The Interplay between m6A RNA Methylation and Noncoding RNA in Cancer. J. Hematol. Oncol. 12 (1), 121. 10.1186/s13045-019-0805-7 31757221 PMC6874823

[B34] Ma X. He Z. Li L. Yang D. Liu G. (2017). Expression Profiles Analysis of Long Non-coding RNAs Identified Novel lncRNA Biomarkers with Predictive Value in Outcome of Cutaneous Melanoma. Oncotarget 8 (44), 77761–77770. 10.18632/oncotarget.20780 29100423 PMC5652813

[B35] Meyer K. D. Saletore Y. Zumbo P. Elemento O. Mason C. E. Jaffrey S. R. (2012). Comprehensive Analysis of mRNA Methylation Reveals Enrichment in 3′ UTRs and Near Stop Codons. Cell 149 (7), 1635–1646. 10.1016/j.cell.2012.05.003 22608085 PMC3383396

[B36] Pan Y. Ma P. Liu Y. Li W. Shu Y. (2018). Multiple Functions of m6A RNA Methylation in Cancer. J. Hematol. Oncol. 11 (1), 48. 10.1186/s13045-018-0590-8 29587823 PMC5870302

[B37] Ramilowski J. A. Yip C. W. Agrawal S. Chang J. C. Ciani Y. Kulakovskiy I. V. (2020). Functional Annotation of Human Long Noncoding RNAs via Molecular Phenotyping. Genome Res. 30 (7), 1060–1072. 10.1101/gr.254219.119 32718982 PMC7397864

[B38] Rong D. Dong Q. Qu H. Deng X. Gao F. Li Q. (2021). m6A-induced LINC00958 Promotes Breast Cancer Tumorigenesis via the miR-378a-3p/YY1 axis. Cell Death Discov. 7 (1), 27. 10.1038/s41420-020-00382-z 33531456 PMC7854648

[B39] Su T. Huang M. Liao J. Lin S. Yu P. Yang J. (2021). Insufficient Radiofrequency Ablation Promotes Hepatocellular Carcinoma Metastasis through M 6 A mRNA Methylation Dependent Mechanism. Hepatology 10.1002/hep.31766 33638162

[B40] Tao L. Mu X. Chen H. Jin D. Zhang R. Zhao Y. (2021). FTO Modifies the m6A Level of MALAT and Promotes Bladder Cancer Progression. Clin. Translational Med. 11 (2), e310. 10.1002/ctm2.310 PMC7851431 33634966

[B41] Wang S. Chai P. Jia R. Jia R. (2018). Novel Insights on m6A RNA Methylation in Tumorigenesis: a Double-Edged Sword. Mol. Cancer 17 (1), 101. 10.1186/s12943-018-0847-4 30031372 PMC6054842

[B42] Wang Y. Li D. Lu J. Chen L. Zhang S. Qi W. (2020). Long Noncoding RNA TTN-AS1 Facilitates Tumorigenesis and Metastasis by Maintaining TTN Expression in Skin Cutaneous Melanoma. Cell Death Dis 11 (8), 664. 10.1038/s41419-020-02895-y 32820147 PMC7441063

[B43] Wei X. Gu X. Ma M. Lou C. (2019). Long Noncoding RNA HCP5 Suppresses Skin Cutaneous Melanoma Development by Regulating RARRES3 Gene Expression via Sponging miR-12. Ott Vol. 12, 6323–6335. 10.2147/OTT.S195796 PMC6698080 31496735

[B44] Yang S. Xu J. Zeng X. (2018). A Six-Long Non-coding RNA Signature Predicts Prognosis in Melanoma Patients. Int. J. Oncol. 52 (4), 1178–1188. 10.3892/ijo.2018.4268 29436619 PMC5843393

[B45] Yao Y. Yang Y. Guo W. Xu L. You M. Zhang Y.-C. (2021). METTL3-dependent m6A Modification Programs T Follicular Helper Cell Differentiation. Nat. Commun. 12 (1), 1333. 10.1038/s41467-021-21594-6 33637761 PMC7910450

[B46] Ye M. Dong S. Hou H. Zhang T. Shen M. (2021). Oncogenic Role of Long Noncoding RNAMALAT1 in Thyroid Cancer Progression through Regulation of the miR-204/IGF2BP2/m6A-MYC Signaling. Mol. Ther. - Nucleic Acids 23, 1–12. 10.1016/j.omtn.2020.09.023 33312756 PMC7711188

[B47] Yu J. Chai P. Xie M. Ge S. Ruan J. Fan X. (2021). Histone Lactylation Drives Oncogenesis by Facilitating m6A Reader Protein YTHDF2 Expression in Ocular Melanoma. Genome Biol. 22 (1), 85. 10.1186/s13059-021-02308-z 33726814 PMC7962360

[B48] Zaccara S. Ries R. J. Jaffrey S. R. (2019). Reading, Writing and Erasing mRNA Methylation. Nat. Rev. Mol. Cel Biol. 20 (10), 608–624. 10.1038/s41580-019-0168-5 31520073

[B49] Zhou J. Wang J. Hong B. Ma K. Xie H. Li L. (2019). Gene Signatures and Prognostic Values of m6A Regulators in Clear Cell Renal Cell Carcinoma - a Retrospective Study Using TCGA Database. Aging 11 (6), 1633–1647. 10.18632/aging.101856 30877265 PMC6461179

[B50] Zhou Y. Zeng P. Li Y.-H. Zhang Z. Cui Q. (2016). SRAMP: Prediction of Mammalian N6-Methyladenosine (m6A) Sites Based on Sequence-Derived Features. Nucleic Acids Res. 44 (10), e91. 10.1093/nar/gkw104 26896799 PMC4889921

[B51] Zhu P. He F. Hou Y. Tu G. Li Q. Jin T. (2021). A Novel Hypoxic Long Noncoding RNA KB-1980E6.3 Maintains Breast Cancer Stem Cell Stemness via Interacting with IGF2BP1 to Facilitate C-Myc mRNA Stability. Oncogene 40, 1609–1627. 10.1038/s41388-020-01638-9 33469161 PMC7932928

