# Supplementary figures and images for: m6A-Related lncRNAs Are Potential Biomarkers for the Prognosis of Metastatic Skin Cutaneous Melanoma
Source: Front Mol Biosci. 2021 May 5;8:687760. doi: 10.3389/fmolb.2021.687760 (PMC8131514; doi:10.3389/fmolb.2021.687760)

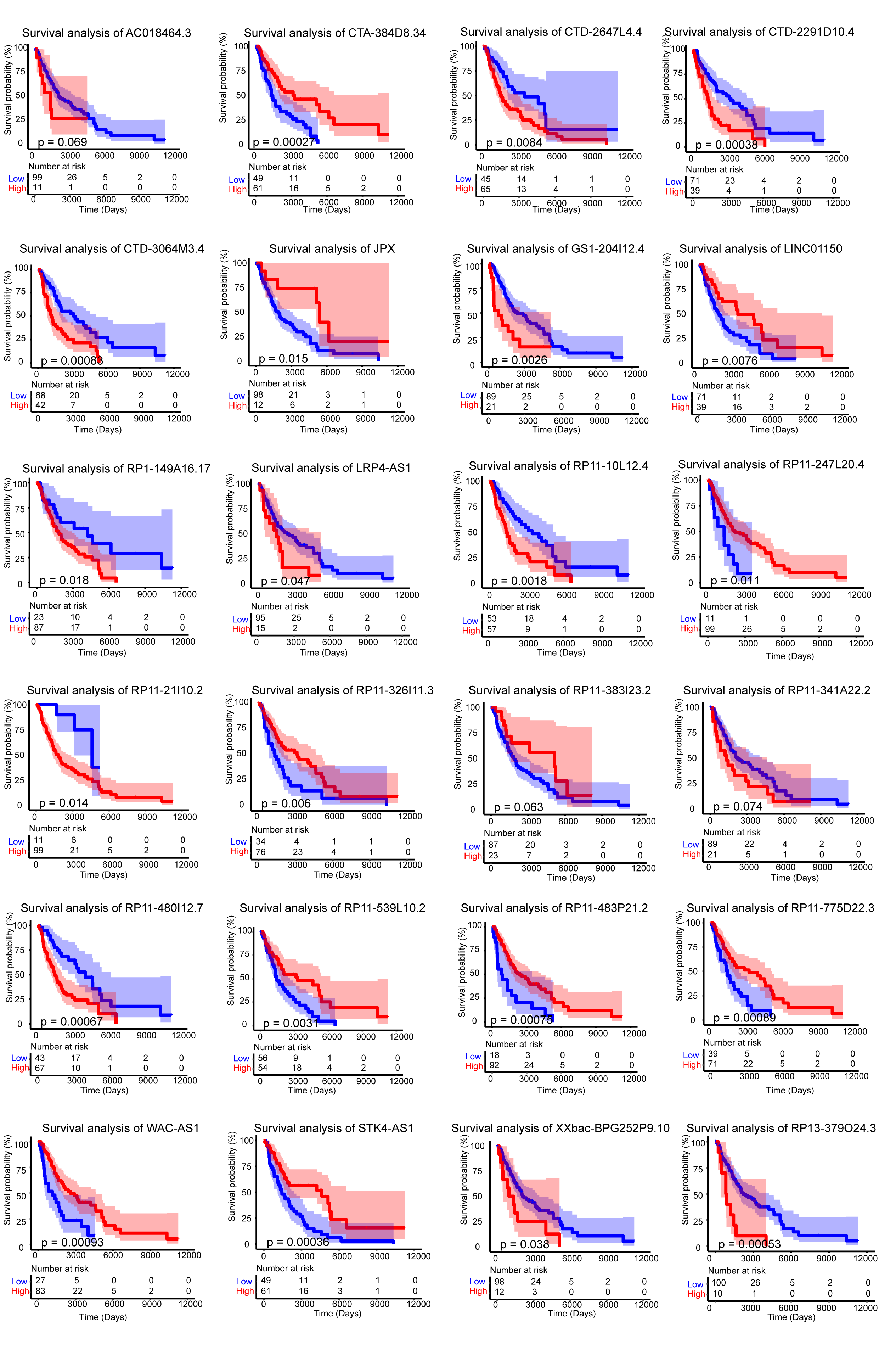

Supplement: Supplementary file 3 [file Image3.TIF]

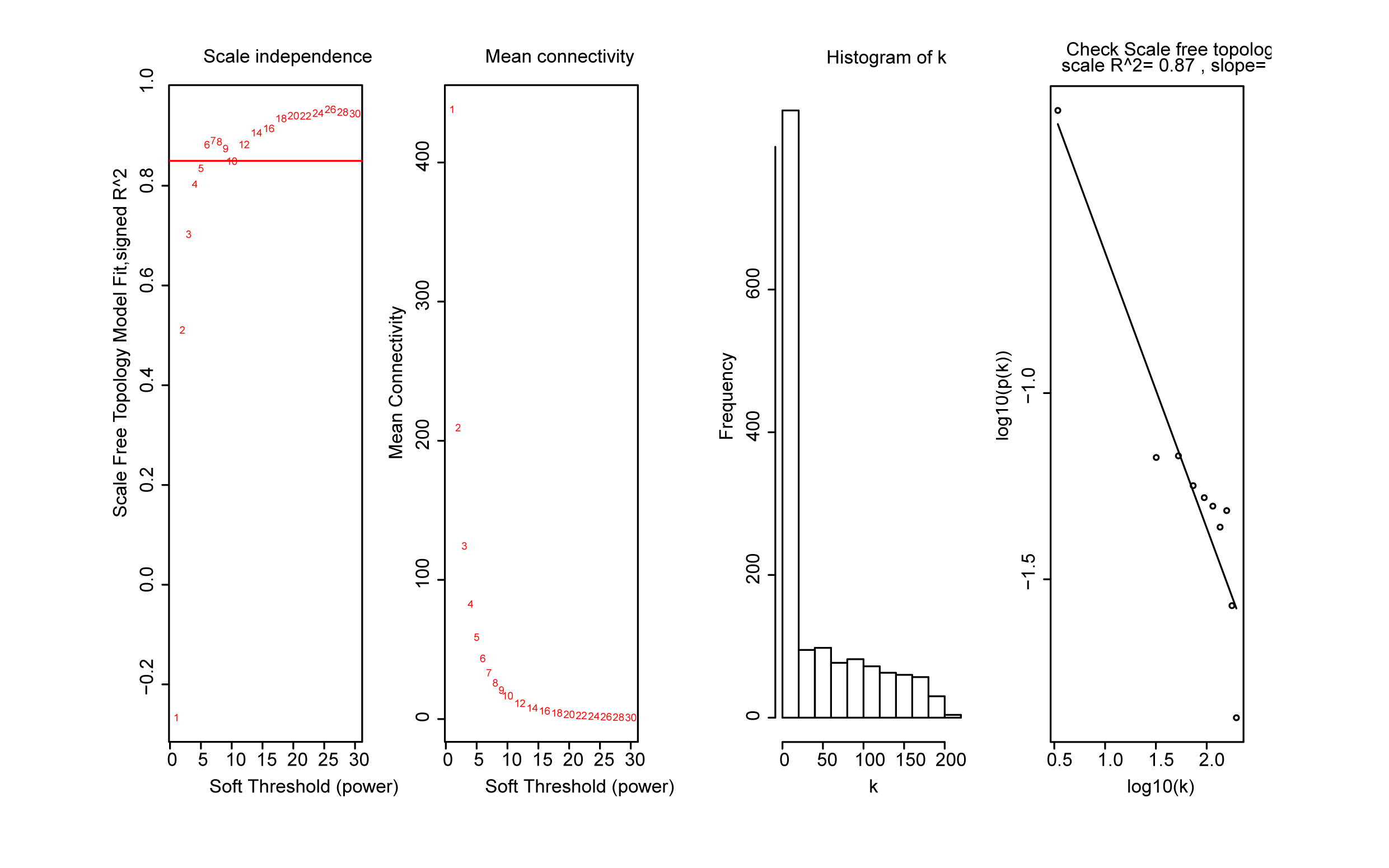

Supplement: Supplementary file 4 [file Image4.TIF]

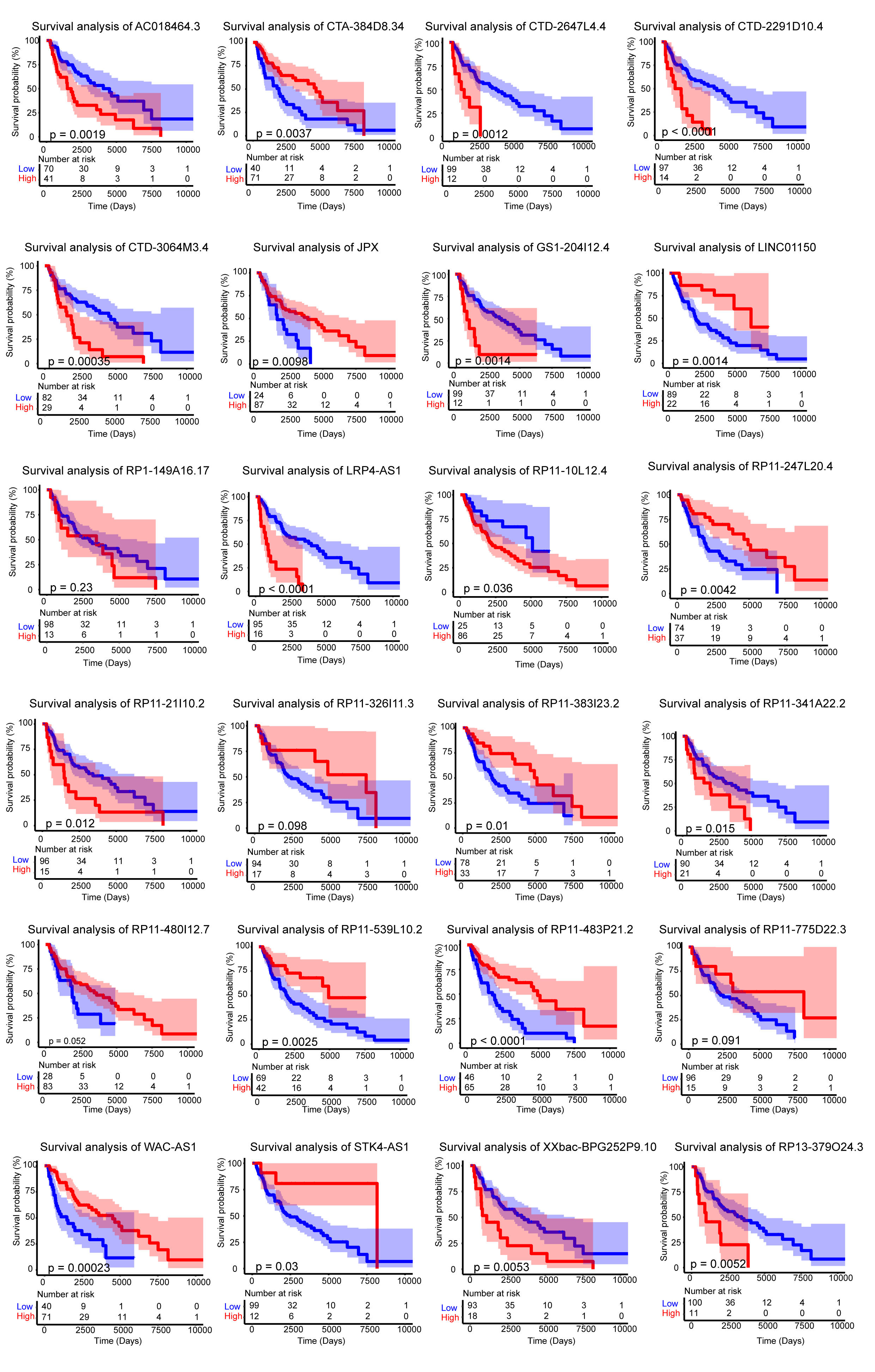

Supplement: Supplementary file 5 [file Image2.TIF]

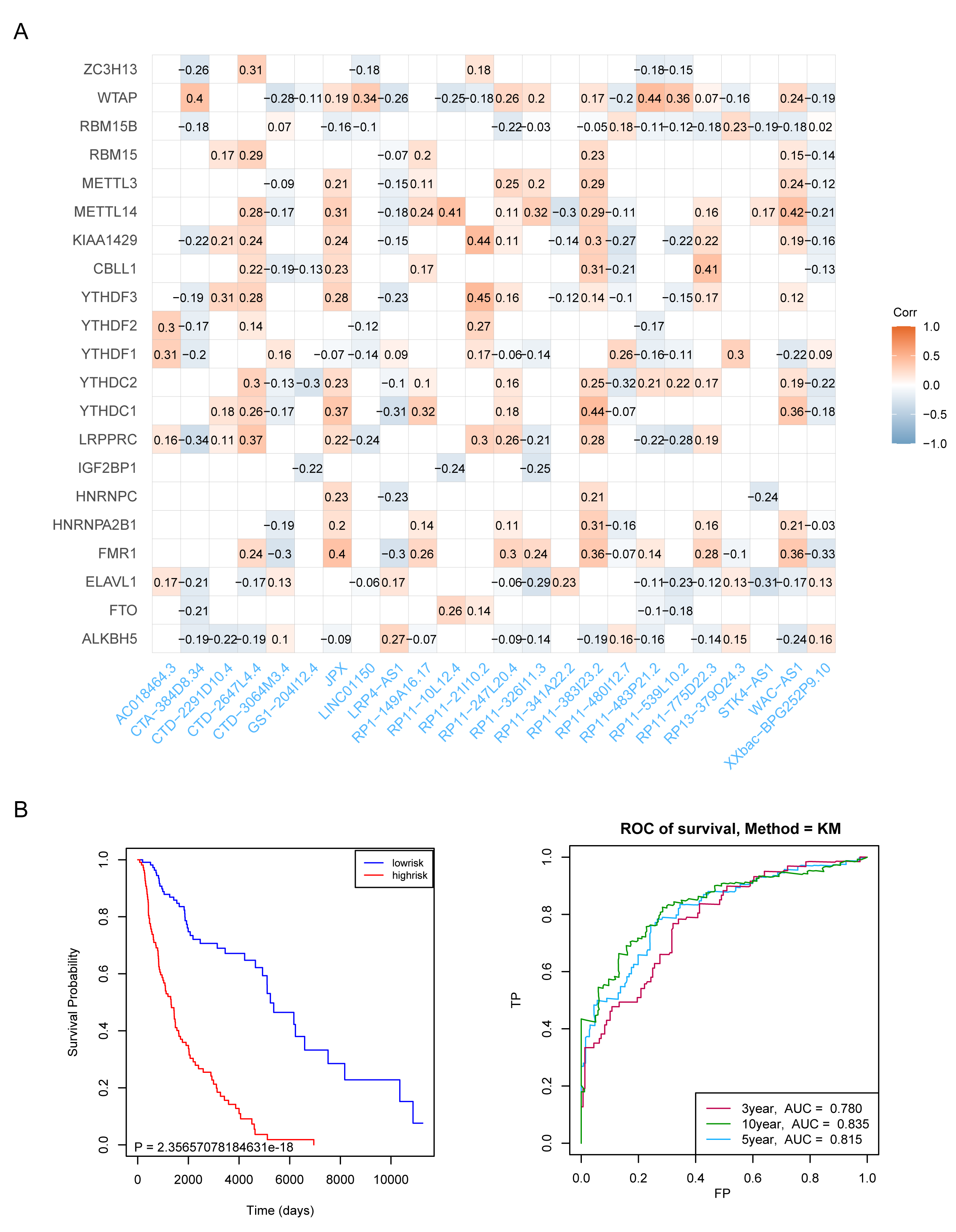

Supplement: Supplementary file 6 [file Image1.TIF]

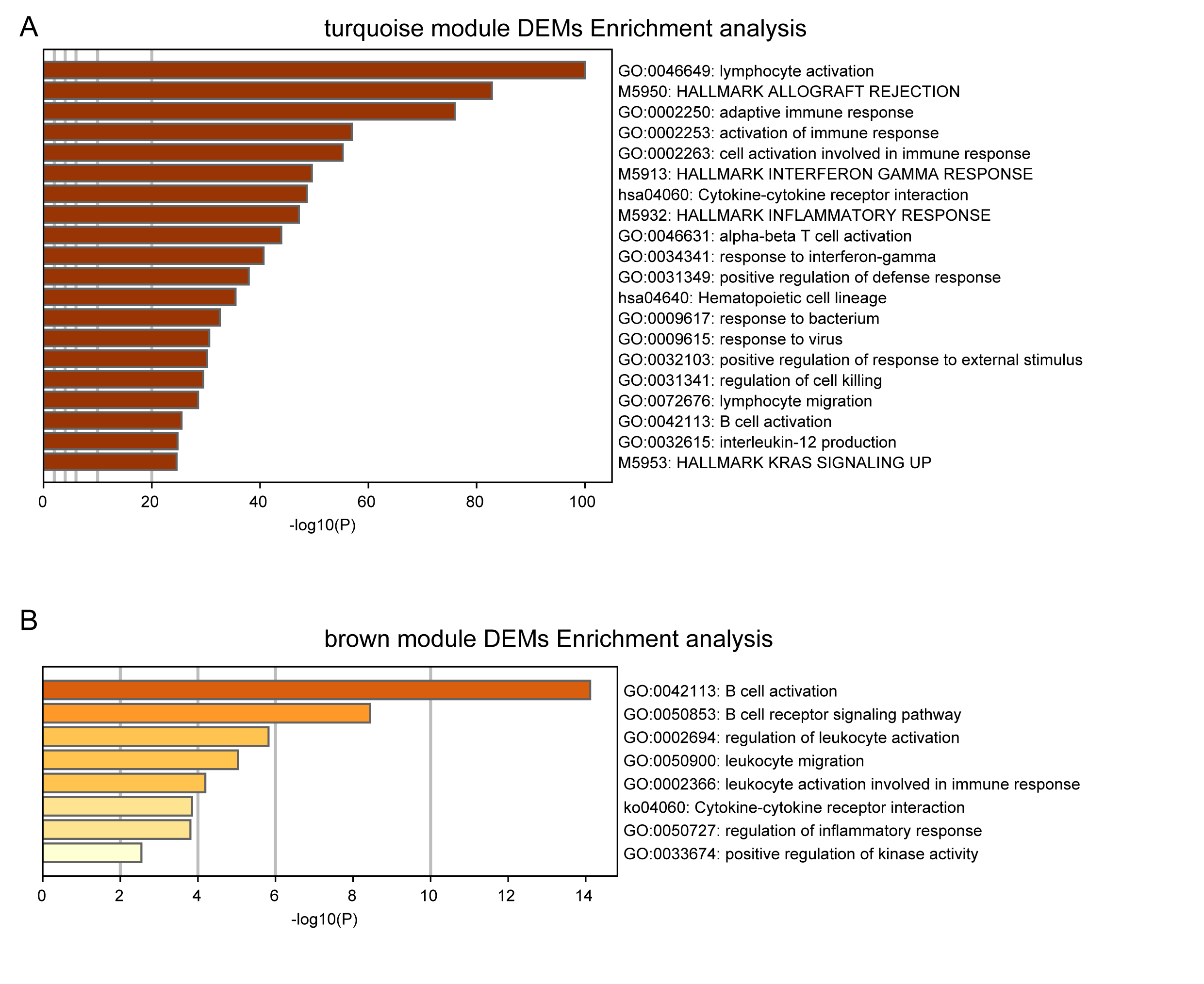

Supplement: Supplementary file 9 [file Image5.TIF]
